# Multi-System-Level Analysis Reveals Differential Expression of Stress Response-Associated Genes in Inflammatory Solar Lentigo

**DOI:** 10.3390/ijms25073973

**Published:** 2024-04-03

**Authors:** Jisu Jeong, Wonmin Lee, Ye-Ah Kim, Yun-Ji Lee, Sohyun Kim, Jaeyeon Shin, Yueun Choi, Jihan Kim, Yoonsung Lee, Man S. Kim, Soon-Hyo Kwon

**Affiliations:** 1 Translational-Transdisciplinary Research Center, Clinical Research Institute, Kyung Hee University Hospital at Gangdong, Kyung Hee University College of Medicine, Seoul 05278, Republic of Korea; symply501@khu.ac.kr (J.J.); dldnjsals345@khu.ac.kr (W.L.); yeak426@gmail.com (Y.-A.K.); sori2013@naver.com (S.K.); jyshin24@khu.ac.kr (J.S.); uag43@khu.ac.kr (Y.C.); kknowing@khu.ac.kr (J.K.); ylee3699@khu.ac.kr (Y.L.); 2 Department of Biomedical Science and Technology, Graduate School, Kyung Hee University, Seoul 02453, Republic of Korea; 3 Department of Medicine, Kyung Hee University College of Medicine, Seoul 02453, Republic of Korea; 4 Department of Dermatology, Kyung Hee University Hospital at Gangdong, Kyung Hee University College of Medicine, Seoul 05278, Republic of Korea; yjlee7799@naver.com; 5 Department of Mathematics, Kyung Hee University College of Science, Seoul 02453, Republic of Korea

**Keywords:** solar lentigo, RNA-seq data, inflammation, oxidative stress

## Abstract

Although the pathogenesis of solar lentigo (SL) involves chronic ultraviolet (UV) exposure, cellular senescence, and upregulated melanogenesis, underlying molecular-level mechanisms associated with SL remain unclear. The aim of this study was to investigate the gene regulatory mechanisms intimately linked to inflammation in SL. Skin samples from patients with SL with or without histological inflammatory features were obtained. RNA-seq data from the samples were analyzed via multiple analysis approaches, including exploration of core inflammatory gene alterations, identifying functional pathways at both transcription and protein levels, comparison of inflammatory module (gene clusters) activation levels, and analyzing correlations between modules. These analyses disclosed specific core genes implicated in oxidative stress, especially the upregulation of nuclear factor kappa B in the inflammatory SLs, while genes associated with protective mechanisms, such as SLC6A9, were highly expressed in the non-inflammatory SLs. For inflammatory modules, Extracellular Immunity and Mitochondrial Innate Immunity were exclusively upregulated in the inflammatory SL. Analysis of protein–protein interactions revealed the significance of CXCR3 upregulation in the pathogenesis of inflammatory SL. In conclusion, the upregulation of stress response-associated genes and inflammatory pathways in response to UV-induced oxidative stress implies their involvement in the pathogenesis of inflammatory SL.

## 1. Introduction

Solar lentigo (senile lentigo, SL) is a commonly acquired pigmentary condition in the elderly that is considered a hallmark of skin photoaging. It appears as solitary or multiple well-defined, light to dark brown colored macules or patches on sun-exposed areas, especially in the face and hand dorsum. The diagnosis of SL is primarily determined by histopathology, which has been reported as two patterns—a flattened epidermis with basal melanosis and epidermal hyperplasia with elongated rete ridges [1]. Other non-invasive diagnostic methods for SL include dermoscopy (moth-eaten border, homogenous pigmentation, pigment network, pseudonetwork, erythema, and bluish–gray granules) [2], reflectance confocal microscopy (suprapapillary cobblestone pattern, polycystic papillary contours, bulbous projections, and crumbled coarse bright collagen) [3], and line-field confocal optical coherence tomography [4].

Pathogenesis of SL involves chronic ultraviolet (UV) exposure, cellular senescence, and upregulated melanogenesis [5]. Chronic exposure to UV induces skin to generate chemical, hormonal, and neural signals such as cytokines, corticotropin-releasing hormone, urocortins, proopiomelanocortin-peptides, and enkephalins, which could be released into circulation to exert systemic effects [6]. Chronic UV exposure is a major cause of pigmentary skin disorder, directly upregulating melanogenesis via the expression of melanocyte-inducing transcription factor (MITF) and melanogenic enzymes such as tyrosinase, tyrosinase-related protein (TRP)-1 in melanocytes [7]. Chronic UV exposure also causes cellular senescence of the skin, especially keratinocytes and fibroblasts. Keratinocytes in SL show decreased proliferation, impaired differentiation, and hyperplasia, all of which implicate cellular senescence [8,9,10]. Immunohistochemical studies revealed the presence of p16*^INK4A^*-positive senescent fibroblasts in the upper dermis of SL [11]. The senescence of fibroblasts causes the alternation of their secretory proteins, such as the stromal cell-derived factor (SDF)-1 and the growth/differentiation factor (GDF)-15 [11,12]. In combination with the disrupted basement membrane, senescence-associated secretory proteins from dermal fibroblasts reach melanocytes to serve as stimuli for melanogenesis [13].

Inflammation is also likely to be involved in the development of SL. In response to UV exposure and stress, neuroendocrine and immune systems are activated via signaling molecules by resident and immune cells and the release of neurotransmitters, endocrine factors, neuropeptides, and cytokines from nerve endings [14]. Disturbance in the neurohormonal mediators and cytokines regulating physiological skin functions results in cutaneous pigmentary disorders [14]. Histologically, inflammatory features such as infiltration of inflammatory cells and interface changes were found in SL [2]. Gene expression profiling showed increased expression of inflammation-related genes in SL [9]. A genome-wide association study reported the association between HLA genes and the severity of SL [15]. Monocyte chemoattractant protein (MCP)-1, a proinflammatory chemokine related to tissue aging, might be involved in the development of SL by inducing senescence of keratinocytes and proliferation of melanocytes [16]. Increased expression of interferon (IFN)-γ and upregulated IFN- γ-stimulated proinflammatory chemokine genes were found in delayed pigmented spots in a mouse model [17]. Higher expression of tumor necrosis factor (TNF)-α and lower expression of interleukin (IL)-1α were found in SL compared to perilesional skin [18]. Although chronic ultraviolet exposure and cellular senescence in combination might cause the inflammatory process during the development of SL, what inflammatory pathway is involved in the development of SL remains highly uncertain.

Several studies have reported the association of “erythematous SL” or “inflammatory SL” with the infiltration of inflammatory cells and interface changes in histology and high susceptibility to post-inflammatory hyperpigmentation after laser treatment [2,19,20]. Morgan et al. demonstrated that the inflammatory SL could be a clinicopathologic spectrum of lichen planus-like keratosis, representing the inflammatory stage of involuting SL [21].

The aim of this study is to provide a considerable understanding of the UV-induced inflammatory processes associated with the development of SL via the identification of their corresponding inflammatory-gene regulatory mechanisms. To investigate the specific underlying gene regulations, we obtained transcription profiles from skin samples of SL via mRNA sequencing and implemented multiple comparative analysis approaches by comparing the following: (i) significantly differentially expressed genes; (ii) collectively associated functional pathways linked to inflammatory gene groups; (iii) relative gene expression profiles of the custom-made core inflammatory genes; (iv) estimated activation levels of inflammatory modules with the core genes; (v) functionally associated clusters based on protein–protein interaction network; (vi) correlations of inflammatory modules with the core genes. Using the various approaches, we examined potential regulatory mechanisms of functionally associated genes or modules via common or different patterns based on the comparison between the inflammatory and non-inflammatory SL.

## 2. Results

### 2.1. Histopathologic Characteristics of SL Samples

To investigate underlying inflammation-associated gene regulation for SL, we analyzed the histopathologic characteristics of SL samples from six patients. Among them, five (83.3%) were female and one (16.7%) was male. The mean age was 80.8 ± 10.6 years (range, 67–96 years). The samples were obtained from the cheek (83.3%) and temple (16.7%).

Table 1 describes the histopathologic characteristics of SL analyzed in this study. All samples had basal hyperpigmentation and solar elastosis, which was consistent with the diagnostic feature of SL. Dermal melanophages were observed in 83.3% of the samples. Epidermal hyperplasia with rete ridge elongation was shown in 33.3% of the samples, while 66.7% showed epidermal flattening. Infiltration of inflammatory cells was observed in five samples (83.3%). Among them, infiltration of inflammatory cells was found to be a moderate-to-severe grade in the three samples, while the other two samples showed a mild grade.

**Figure 1:**
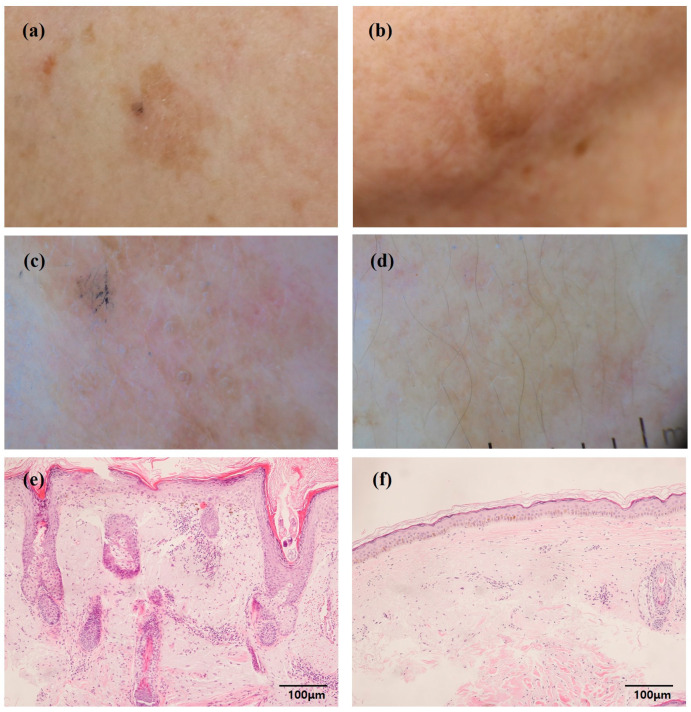
Clinical, dermoscopic, and histopathological characteristics of inflammatory and non-inflammatory solar lentigines (SLs). (**a**,**c**,**e**) Inflammatory SL. (**b**,**d**,**f**) Non-inflammatory SL.

Based on the result of the histopathologic study, SL samples were divided into two categories for downstream analyses: the inflammatory SLs showing moderate-to-severe infiltration of inflammatory cells; and the non-inflammatory SLs showing none or mild infiltration of inflammatory cells (Figure 1) [20].

### 2.2. Inflammation-Associated Genes Upregulated in the Inflammatory SL

**Figure 2:**
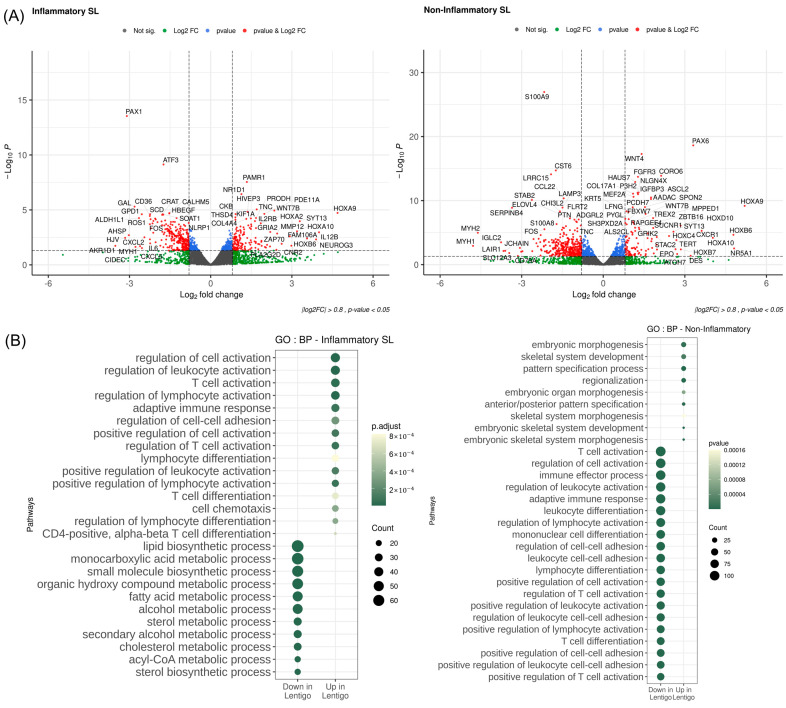
Differentially expressed genes (DEGs)and enriched pathways. (**A**) Volcano plot of DEGs between the inflammatory and non-inflammatory solar lentigines (SLs). The genes were collected using the keyword “inflammation” within the Human Gene Sets of MsigDB. The *X*-axis represents log2 fold change, and the *Y*-axis represents the log10 of Wald test *p*-value by DESeq2 (*p*-value < 0.05, log2 fold-change > 0.8). (**B**) Dot plot of gene ontology (GO) analysis of Biological Processes. The analysis utilized genes associated with inflammation (*p*-value < 0.05, absolute log2 fold-change < 0.5). The *Y*-axis represents GO terms, and the *X*-axis represents upregulation or downregulation in solar lentigo. The dot sizes correspond to the size of the gene set associated with each pathway. The color gradient is proportional to the adjusted *p*-value (Benjamini–Hochberg). BP, Biological Processes; GO, gene ontology; SL, solar lentigo.

To search for condition-specific and differential characteristics SLs at the transcription level, we implemented typical differential analyses, including GO analysis using inflammation-associated genes. As depicted in Figure 2A, we identified a sufficient number of differentially expressed genes (DEGs) from both inflammatory and non-inflammatory SLs when compared to adjacent normal skin, where the DEGs displayed distinct variations across samples of the two groups. The comparison between the inflammatory SLs and adjacent normal skin highlighted potential links with inflammatory responses.

In addition, we explored inflammatory-associated pathways via GO analysis using the DEGs of inflammation-associated genes selectively obtained from MSigDB (Figure 2B). While the significance of upregulation in the non-inflammatory SL was relatively inconsiderable, upregulation of the inflammatory SL indicated interconnections with several inflammation-associated activation regulations, such as the regulation of leukocyte activation, regulation of lymphocyte activation, and regulation of T-cell activation. On the other hand, downregulation of the inflammatory SL was associated with several different metabolic processes, including the monocarboxylic acid metabolic process.

### 2.3. Core Inflammatory Genes Revealed Significant Alterations on the Inflammatory Responses to Oxidative Stress in the Inflammatory SL

**Figure 3:**
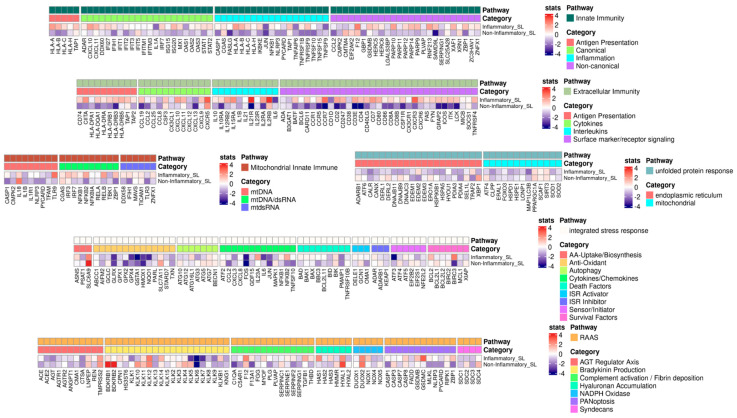
Heatmap of the custom-made core inflammatory genes with Wald-test statistics comparing the inflammatory and non-inflammatory solar lentigines (SLs). The core gene list was curated by Guarnieri et al. [22]. Upregulated genes are indicated in red, and downregulated genes are indicated in blue. ISR, integrated stress response; SL, solar lentigo.

To identify a potential gene list of SL-specific inflammatory responses, we explored specific gene expression alterations between the inflammatory and non-inflammatory SLs. In this analysis (Figure 3), we specifically referenced the custom-made core-inflammatory-response gene list from Guarnieri et al. [22]. Several genes from the core list were upregulated in the inflammatory SL. Specifically, within Extracellular Immunity, we detected the highest upregulation of genes, including IL21R, CCR7, CD3E, IL15RA, CD7, CXCL10, IL12RB2, CD4, ICOS, CSF1R, CD86, and CXCR3, while genes in Innate Immunity exhibited upregulation, including SAMD9L, TNFRSF1B, ZBP1, IL1A, and XAF1. Additionally, genes such as GSDMC, TGFB1, ZBP1, and HYAL1 in RAAS were identified, while we noted genes in Integrated Stress Response (ISR), such as IL23A, NFkB2, and SLC6A9. Lastly, genes in Mitochondrial Innate Immunity encompassed TLR9, HAS1, NFkB2, and ZBP1.

From another perspective, we looked into whether certain genes are intimately associated with UV-induced damage or oxidative stress responses. In particular, the upregulation of nuclear factor kappa B (NF-κB) is linked to prolonged UV exposure within the inflammatory SL. Additionally, upregulation of genes (i.e., CXCR3, XAF1, and ZBP1) was also detected in the inflammatory SL. Conversely, highly expressed genes in the non-inflammatory SL appeared to be implicated in protective mechanisms against UV-induced damage, including SLC6A9 and HYAL.

### 2.4. Inflammatory Systems Revealed Different Alteration Patterns between the Inflammatory and Non-Inflammatory SLs

**Figure 4:**
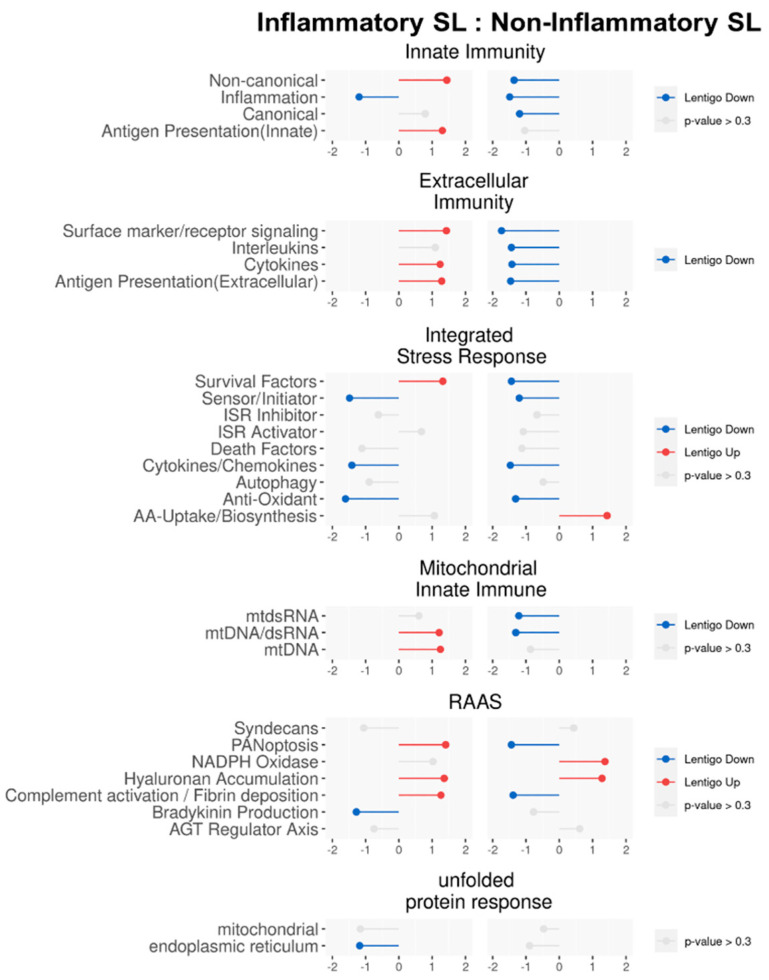
Lollipop plot of pathway enrichment analysis by fast Gene Set Enrichment Analysis (fGSEA) where the pathways were customized by Guarnieri et al. [22]. The length of the stick is the degree of nominal enrichment score (NES). Grey means that it is not significantly enriched (*p*-value > 0.3), while red indicates upregulation, and blue indicates downregulation in solar lentigo (SL). ISR, integrated stress response; RAAS, Renin–Angiotensin–Aldosterone System; SL, solar lentigo.

To evaluate how activation levels of inflammatory systems differ dynamically between the inflammatory and non-inflammatory SLs, we compared their enrichment scores for each inflammatory module (i.e., gene clusters) by computing the corresponding NES via fGSEA [23]. Overall, as depicted in Figure 4, differential activation patterns of inflammatory modules were uncovered between the inflammatory and non-inflammatory SLs in the following systems: Innate Immunity; Extracellular Immunity; Mitochondrial Innate Immunity; and RAAS. However, no significant differences were shown in the ISR and Unfolded Protein Response (UPR). In other words, while the gene expression profile patterns associated with the core inflammatory responses differed between the inflammatory and non-inflammatory SLs, the patterns associated with cellular stresses, including oxidative stress, appeared to be similar across both patient groups.

Different activation patterns of inflammatory modules distinguished the inflammatory SL from the non-inflammatory SL in the four systems. Specifically, within Innate Immunity (both canonical and non-canonical pathways), upregulations were observed in the inflammatory SL, while downregulation occurred in the non-inflammatory SL. Considering the two modules, which comprise a majority of IFN-stimulated genes (ISGs), the initial phases of innate immune responses against invading pathogens via the utilization of reactive oxygen species (ROS) can be considered [24]. All four modules in Extracellular Immunity (Surface marker/Receptor signaling, Interleukins, Cytokines, and Antigen Presentation) unveiled opposite patterns, where upregulations in the inflammatory SL and downregulations in the non-inflammatory SL were shown. While PANoptosis and Complement activation/Fibrin deposition in the Renin–Angiotensin–Aldosterone System (RAAS) were upregulated in the inflammatory SL and downregulated in the non-inflammatory SL, Hyaluronan Accumulation was upregulated in both groups, reflecting chronic UV-induced degradation of dermal hyaluronic acid [25]. For Mitochondrial Innate Immunity, all the modules, such as mtdsRNA, mtDNA/dsRNA, and mtDNA, exhibited opposite patterns—upregulations in the inflammatory SL and downregulations in the non-inflammatory SL. The alterations in the Mitochondrial Innate Immunity could be significant for antibacterial immunity by generating ROS or activating innate immune responses leading to cellular damage and stress [26].

For ISR, dynamics can depend on responses to a range of physiological changes and various pathological conditions (i.e., hypoxia, amino acid deprivation, glucose deprivation, and viral infection) [27]. Three ISR modules, such as Sensor/Initiator, Cytokines/Chemokines, and Antioxidant, were downregulated in both the inflammatory and non-inflammatory SLs. This may suggest that cellular damages via oxidative stress and their corresponding repair/rebuilding systems could be relatively deactivated for both the inflammatory and non-inflammatory SLs. Along with ISR, UPR, which is associated with immune cell functions, innate immune signaling, and managing oxidative stress within the endoplasmic reticulum, was also downregulated in both the inflammatory and non-inflammatory SL [28].

### 2.5. Protein–Protein Interaction-Based Clusters Disclosed Alterations Regarding Inflammatory Responses

**Figure 5:**
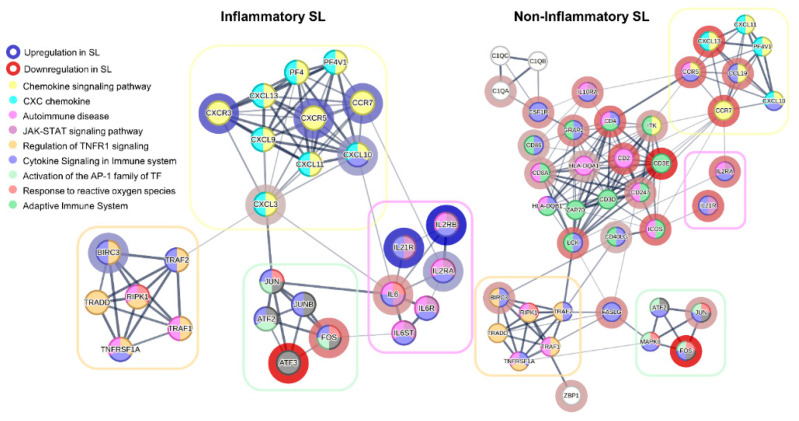
Cluster visualization based on protein–protein interaction (PPI) networks, utilizing differentially expressed genes (DEGs) in solar lentigo (SL) using STRING database. DEGs were selected 32 high-ranked genes for the inflammatory SL and 36 genes for the non-inflammatory SL. Interactions with protein association confidence scores above 0.4 included setting the number to 10 in the first shell and 5 in the second shell. The color of the nodes represents pathways that have been functionally enriched; the border in red indicates downregulated genes, and the border in blue indicates upregulated genes. SL, solar lentigo.

To search for potential functional clusters at the protein level, we explored potential associations between corresponding proteins of the DEGs via the protein–protein interaction (PPI) network based on STRING [29]. As depicted in Figure 5, four functional clusters that were common to both the inflammatory SL and the non-inflammatory SL were detected: Chemokine Signaling Pathway; Autoimmune Disease; Regulation of TNFR1 Signaling; and Activation of the AP-1 Family of Transcription Factor. While the two clusters—Regulation of TNFR1 Signaling and Activation of the AP-1 Family of Transcription Factor—were nearly the same between the two groups, the other two clusters—Chemokine Signaling Pathway and Autoimmune Disease—demonstrated bigger clusters in the inflammatory SL compared with non-inflammatory SL.

Considering the identification of potential PPI clusters shedding light on underlying mechanisms of SL-specific inflammatory responses, we consistently observed suppression of JUN, FOS, and ATF3 in both the inflammatory and non-inflammatory SLs. This underscores the potential role of the AP-1 complex in the pathogenesis of SL. We also detected that clusters involved in the CXC chemokine and chemokine signaling pathways were upregulated in the inflammatory SL but downregulated in the non-inflammatory SL. This finding emphasizes the significance of CXCR3 upregulation in the pathogenesis of inflammatory SL. Additionally, we identified a similar pattern in the JAK-STAT signaling pathway, which appears to be influenced by these chemokines. The chemokine activity may drive the activation of JAK-STAT signaling in the inflammatory SL while suppressed in the non-inflammatory SL.

### 2.6. Different Correlation Patterns of Inflammatory Systems between the Inflammatory and Non-Inflammatory SLs

**Figure 6:**
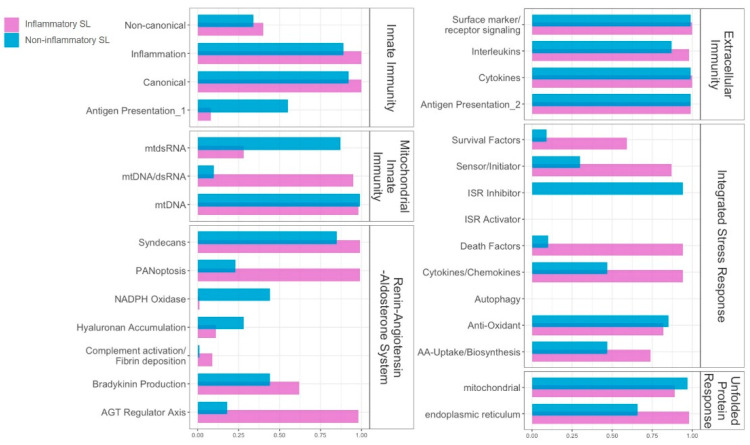
Pearson’s correlations between modules of the inflammatory systems for inflammatory and non-inflammatory solar lentigines (SLs). The correlations estimated by Q-Lattice with nominal enrichment score (NES) values of modules and their inflammatory systems were visualized. Each NES was computed with the core genes of both inflammatory SL (*p*-value < 0.3) and non-inflammatory SL (*p*-value < 0.3). SL, solar lentigo.

While recognizing inflammatory modules differentially activated between the inflammatory and non-inflammatory SLs, we searched for correlation patterns between the modules or with their corresponding systems, where correlation coefficients were computed utilizing QLattice [30] (Figure 6). The comparative analysis revealed high correlation patterns (i.e., higher than 0.8) between modules from the inflammatory and non-inflammatory SLs, including (i) Innate Immunity and its corresponding two modules such as Inflammation and Canonical, (ii) Extracellular Immunity and all its corresponding modules, (iii) Mitochondrial Innate Immunity and its module, mtDNA, (iv) ISR and one of its modules such as Anti-Oxidant, (v) RAAS and its module such as Syndecans, (vi) UPR and its modules such as Mitochondrial module. Interestingly, Extracellular Immunity and UPR displayed higher correlations with their corresponding modules, suggesting that these modules were coordinately regulated in SLs compared to adjacent normal skin.

On the other hand, modules with higher correlations of the inflammatory SL and lower correlations of the non-inflammatory SL (with differences > 0.6) include (i) mtDNA/dsRNA in Mitochondrial Innate Immunity, (ii) Survival factors, Sensor/initiator, and Death factors in ISR, and (iii) PANoptosis and AGT regulator axis in RAAS. Conversely, modules with higher correlations in the non-inflammatory SL and lower correlations in the inflammatory SL encompass (i) Antigen Presentation in Innate Immunity, (ii) mtdsRNA in Mitochondrial Innate Immunity, (iii) NADPH Oxidase in RAAS, and (iv) ISR Inhibitor in ISR. This suggests a potential for more coordinated module regulations on the corresponding inflammatory systems in the inflammatory SL than the non-inflammatory SL, while there are fewer coordinated modules between the inflammatory and non-inflammatory SLs in ISR and RAAS.

## 3. Discussion

This study investigated the underlying genes that regulate cellular processes intimately associated with SL-specific inflammatory responses. To capture the dynamics of gene expressions specific to SL, we analyzed RNA-seq data from SL samples with or without the inflammatory characteristics in histopathology. Different analysis approaches considering multi-system levels (i.e., transcription and protein levels) encompassed detecting functional pathways linked to GO-associated database, discovering gene expression alterations of the core inflammatory gene list, evaluating activation levels of the core inflammatory modules, identifying PPI-based inflammatory-associated clusters, and computing correlation coefficients of the core inflammatory modules.

Since alterations of inflammatory-specific gene expressions were the primary interest, we focused on gene expression profile changes between the inflammatory and non-inflammatory SLs from the custom-made core inflammatory gene list [22], along with the inflammatory-associated gene list from MSigDB. Several genes exhibiting heightened expression in the inflammatory SL, including IL15RA in Extracellular Immunity, might indicate impaired regulation of SL-associated skin inflammation by suppressing key inflammation-associated pathways and modulating T-cell proliferation [31]. Other genes, such as IL-21R, GSDMC, CCR7, TLR9, HAS1/3, IL-17A, IL-22, and CXCL10, have been previously identified as genes associated with various inflammatory skin disorders. IL-21R in Extracellular Immunity, playing a pivotal role in atopic dermatitis by enhancing allergic immune responses with the promotion of migration of dendritic cells toward draining lymph nodes, displayed elevated expressions in various acute skin lesions [32]. In psoriatic lesions, the remarkable upregulation of GSDMC in RAAS suggested its direct involvement in the local disease pathology, potentially via mechanisms associated with inflammatory cell death [33]. CCR7 in Extracellular Immunity was reported to facilitate dendritic cell migration to lymphoid organs [34] and was found to be instrumental in the formation of inducible skin-associated lymphoid tissue within psoriatic lesions [35]. TLR9 in Mitochondrial Innate Immunity, activated in several autoimmune diseases, including psoriasis, is currently under investigation as a potential therapeutic target, where TLR antagonists are being developed for the treatment of these conditions [36]. Hyaluronan synthase subtypes HAS1 and HAS3 in RAAS exhibited distinct roles in skin biology, where HAS1 was associated with regular differentiation and repair processes, while HAS3 was linked to inflammatory responses, particularly in disorders like atopic dermatitis [37]. Both dendritic cells and keratinocytes in psoriasis revealed an overproduction of IL-23 in Extracellular Immunity, subsequently activating Th17 cells in the dermis to generate IL-17A and IL-22 [38]. Previous studies emphasized that CXCL10 in Extracellular Immunity disclosed a critical role in disease development, as it facilitated the recruitment and activation of skin natural killer cells within psoriatic lesions [39].

Considering the activation of inflammatory modules (i.e., gene clusters), Extracellular Immunity revealed the most outstanding differences between the inflammatory and non-inflammatory SLs. Mitochondrial Innate Immunity with upregulation in the inflammatory SL also demonstrated opposing patterns compared with the non-inflammatory SL, implying their potential roles in managing ROS and mitigating cellular damages and stresses. Moreover, in Innate Immunity, a substantial portion of both canonical and non-canonical pathways experienced upregulation in the inflammatory SL, suggesting their involvement in the initial immune response mediated by ROS. Moreover, in terms of associations at the protein level based on PPIs, the upregulation of chemokines emerged as key players in the inflammatory SL, while Cytokine in Extracellular Immunity and Cytokines/Chemokines in ISR disclosed stronger correlations for the inflammatory SL. This finding may imply that chemokines are considerably involved in inflammatory processes and cellular stresses are involved in inflammatory SL cases.

From another perspective, with the inflammatory-associated gene list from MSigDB, GO analysis divulged multiple functional pathways associated with T-cell response, indicating a substantial involvement of these cells in the inflammatory SL. The non-inflammatory SL unveiled an increase in embryonic morphogenesis and skeletal system development, with a general suppression of metabolic processes, contrasting with the inflammatory SL. In spite of the overall decrease in metabolic processes, the elevation of inflammation response in the inflammatory SL suggests that the modulation of inflammation could be critical in the pathogenesis and progression of SL.

GO and other analysis approaches in this study also demonstrated the role of T cells and other immune-associated cells in the pathogenesis of SL. Using changes in gene expression profiling, we identified key players associated with strong inflammation, including CD7, IL23A, CXCL10, CD4, and CSF1R, predominantly implicated in T-cell activation and migration. Additionally, we discovered elevated expressions of NLRP3, CD80, and IL2RA, which are vital for T-cell function. This observation turned out to be in line with the module-correlation analysis by detecting a robust correlation between Surface marker/Receptor signaling, Interleukins, Cytokines, and Antigen Presentation in Extracellular Immunity of the inflammatory SL, providing strong evidence for the involvement of T cells.

Furthermore, the multiple analysis approaches revealed the involvement of the regulatory mechanism of UV-induced oxidative stress and tissue damage in the pathogenesis of SL. Chronic UV exposure is a key environmental risk factor for the development of pigmentary skin disorders. Specifically, in melasma, an imbalance between oxidants and antioxidants necessitates heightened oxidative stress [40]. Some of the genes were implicated in the oxidative response as master regulators within the inflammatory pathway, including NF-κB [41]. Oxidative stress necessitated by UVA radiation could trigger the activation of NF-κB in human skin fibroblasts [42]. It is possible that melanin production is initiated by the upregulation of CXCR3-mediated signaling in Extracellular Immunity, which can also be activated by UVB exposure [43]. Activation of XAF1 in Innate Immunity in SL patients may contribute to the dysfunctional apoptosis process in response to cellular stress [44]. Additionally, while ZBP1 in Innate Immunity is known to be associated with antiviral immunity in regulating oxidative stress in the retina, it is, however, unclear in the context of skin inflammation [45].

Conversely, highly expressed genes in the non-inflammatory SL appeared to be involved in protective mechanisms against UV-induced damage. SLC6A9 encoding a glycine transporter could play a crucial role in keratinocyte responses to UVB exposure since overexpression of SLC6A9 was reported to protect against UVB-induced senescence [46]. HYAL in RAAS, known for promoting wound healing, may offer advantages in wound treatment [47]. Downregulation of these genes in the inflammatory SL might indicate a disruption in cellular regulatory mechanisms against oxidative stress. This suggests that heightened inflammation appears to render SL patients more susceptible to UV-induced oxidative stress, and the sensitivity may trigger downstream pathway signaling. Therefore, the disruption of protective mechanisms could either lead to uncontrolled inflammatory responses or produce an opposing effect, necessitating further investigation.

Considering activation levels of specific inflammatory modules, it appeared that ISR and UPR for both the inflammatory and non-inflammatory SLs had similar patterns with regard to cellular stresses, such as oxidative stress. This observation implies that perturbations in the regulatory mechanisms controlling ROS may significantly contribute to the initial stage of SL development, potentially leading to reduced activation of cellular damage and subsequent repair systems in affected skin [27]. Moreover, additional genes potentially involved in oxidative stress-associated regulatory mechanisms were found in the inflammatory SL.

The limitation of this study includes the small number of skin samples.

## 4. Materials and Methods

### 4.1. Sample Collection

Skin samples (diameter, 5 mm) were obtained from lesional and adjacent normal areas from six patients diagnosed with facial SL. Hematoxylin and eosin stains were performed using part of lesional skin samples (usually diameter, 2 mm) for histopathologic study. The histologic was reviewed by two dermatologists (Y.-J.L. and S.-H.K.) and confirmed by one dermatopathologist. The remaining lesional and adjacent normal skin samples were used for mRNA sequencing. This study was conducted in accordance with the Declaration of Helsinki and the International Conference on Harmonization and Good Clinical Practice Guidelines and was reviewed and approved by the Institutional Review Board of Kyung Hee University Hospital at Gangdong (KHNMC 2022-04-014). Written informed consent was obtained from all participants before enrollment in this study.

### 4.2. Library Construction

Total RNA concentration was determined using Quant-IT RiboGreen assay, where DV200 (% of RNA fragments >200 bp) value was considered for RNA sample quality. For library construction, 100 ng of total RNA was initially fragmented into smaller pieces and reverse-transcribed into first-strand cDNA, followed by second-strand cDNA synthesis, whose products were purified and enriched using PCR to create the cDNA library. To capture the human exonic regions, the standard Agilent SureSelect Target Enrichment protocol was applied. A total of 250 ng of cDNA library, mixed with hybridization buffers, blocking mixes, RNase block, and 5 µL of the SureSelect all exon capture library, underwent washing and a second round of PCR amplification. The purified product was quantified using KAPA Library Quantification kits for Illumina sequencing platforms whose library quality was assessed using the TapeStation D1000 ScreenTape, and Indexed libraries were subsequently submitted for paired-end (2 × 100 bp) sequencing on an Illumina NovaSeq (Illumina, Inc., San Diego, CA, USA).

### 4.3. RNA-Seq Preprocessing

We acquired gene expression profiles from fourteen independent libraries of seven patients with SL, where each patient provided two distinct samples (i.e., SL and control). The paired-end sequencing reads, generated from the Illumina sequencing NovaSeq platform, quality control was processed using Trimmomatic v0.38 by removing adapter sequences and trimming bases with poor base quality. We performed the alignment step with STAR (v2.7.3a) [48] and HTSeq-count (v0.12.4) [49], where RNA-seq reads of the fourteen libraries were mapped to the reference genome, GRCh38, along with its annotation. Having gene expression levels as counts, we applied the normalization step using the DESeq2 package [50] with VST (Variance Stabilizing Transformation).

### 4.4. Analysis of Inflammatory Regulation

After all the preprocessing, we proceeded with typical differential analysis and gene ontology (GO) analysis by applying R packages such as EnhancedVolcano [51] and clusterProfiler [52] using inflammation-associated genes from the GO database. In order to selectively explore inflammatory-specific gene expression patterns, we also adopted the custom-made gene list of inflammatory responses from [22], where Wald-test statistics were applied. To estimate activity levels of modules/systems, a pathway enrichment method, a fast Gene Set Enrichment Analysis (fGSEA) [23], with its Nominal Enrichment Score (NES), was implemented. To search for interactions at the protein level, protein–protein interaction (PPI) networks were generated by STRING [24] using the inflammation-associated DEGs.

### 4.5. Analysis of Module Correlation

We employed QLattice [25], a machine learning-based regression and classification tool, to perform correlation analysis between inflammatory systems and their associated modules. The analysis revealed regression models, with an implicit consideration of Pearson’s correlation using NES values for inflammatory systems and their modules with inflammatory genes filtered by a *p*-value < 0.5 from TPM (Transcript Per Million) normalized expression levels. The calculation was processed by comparing each SL sample with all the normal cases, as well as all the SL samples with all the normal cases. Subsequently, the NES values of inflammatory modules were used as inputs to regression models in QLattice, with the NES values of their corresponding inflammatory systems as outputs.

## 5. Conclusions

In conclusion, the comparison between the inflammatory and non-inflammatory SLs at the multi-system levels (i.e., transcription and protein level) revealed both commonalities and differences in inflammatory-associated responses. To investigate underlying functional mechanisms associated with these factors depending on the inflammation, multiple analysis approaches from different perspectives were extensively executed. Our findings associated with inflammatory responses from the implementation unveiled important fundamental roles in the pathogenesis, development, or progression of SL. Furthermore, considering the cellular stresses induced by the factors, the upregulation of stress response-associated genes in response to UV and oxidative stress implies their involvement in the progression of SL.

## Figures and Tables

**Table 1:** Histopathological characteristics of the solar lentigo samples.

Histologic Features (*n,%*)	Basal Cell Hyperpigmentation	Dermal Melanophages	Solar Elastosis	Rete Ridge Elongation	Infiltration of Inflammatory Cells
Present	6 (100%)	5 (83.3%)	6 (100%)	2 (33.3%)	5 (83.3%)
Absent	0 (0%)	1 (16.7%)	0 (0%)	4 (66.7%)	1 (16.7%)

## Data Availability

The data presented in this study are available on request from the corresponding author.
